# Allogeneic hematopoietic cell transplantation for therapy-related hematological malignancies in patients with multiple myeloma

**DOI:** 10.3389/fonc.2026.1887912

**Published:** 2026-09-25

**Authors:** Markus Maulhardt, Alexander Casimir Angleitner, Judith Büntzel, Gina Westhofen, Margarete Elisabeth Laage, Justin Hasenkamp, Detlef Thomas Haase, Hannes Treiber, Enver Aydilek, Anna Lena Illert, Wolfram Jung, Gerald Georg Wulf

**Affiliations:** 1 Department of Hematology and Medical Oncology, University Medical Center Goettingen, Goettingen, Germany; 2 Evangelisches Klinikum Bethel, University Hospital Bielefeld, Bielefeld, Germany

**Keywords:** acute leukemia, allo-HCT, auto-HCT, multiple myeloma, secondary malignancies, therapy-related hematological malignancies

## Abstract

Improved survival in multiple myeloma (MM) patients after high-dose chemotherapy, autologous hematopoietic cell transplantation (auto-HCT), and novel agents has led to an increasing incidence of therapy-related hematological malignancies (t-HM), although optimal management remains unclear, particularly the role of allogeneic HCT (allo-HCT). We conducted a retrospective single-center study of adult MM patients who underwent first auto-HCT between 2010 and 2025 and subsequently developed t-HM, analyzing clinical, molecular, cytogenetic features, treatment strategies, and outcomes, with a focus on allo-HCT. Among 519 patients, 23 (4.4%) developed t-HM, including therapy-related acute myeloid leukemia (43%), myelodysplastic syndrome (35%), and B-cell acute lymphoblastic leukemia (17%), with a median latency of 58.7 months after auto-HCT. High-risk genetics were common, including *TP53* mutations in 52% and complex cytogenetics in 35%. Fourteen patients (61%) underwent allo-HCT, mainly with reduced-intensity conditioning. Median overall survival (OS) for the cohort was 31.5 months, while patients receiving allo-HCT had significantly superior OS compared with non-transplanted patients (median not reached vs. 2.5 months; *P* = 0.046). Therapy-related hematologic malignancies after MM treatment are uncommon but aggressive and genetically high-risk. In this cohort, allo-HCT was associated with improved survival and may represent a potentially curative approach in selected patients despite substantial toxicity.

## Introduction

Bortezomib-based induction therapy and lenalidomide maintenance after high dose melphalan conditioning followed by autologous hematopoietic cell transplantation (auto-HCT) are standard of care for eligible patients with multiple myeloma (MM), the second most common hematological malignancy in Western countries (1–5). Prolonged duration of survivorship in relapse through improved subsequent therapy lines including novel, targeted agents like chimeric antigen receptor (CAR) T-cells or bispecific antibodies place MM patients at increased risk of encountering possible long-term adverse events of exposure to cytotoxic chemotherapy (6, 7). Specifically, these patients exhibit an elevated risk of therapy-related hematological malignancies (t-HM) like acute myeloid leukemia and myelodysplastic syndrome (t-AML/MDS), B-cell acute lymphoblastic leukemia (B-ALL) and other second primary malignancies (SPM) (6, 8). Typical myeloma-directed agents in this context are alkylating agents, topoisomerase II inhibitors, anti-metabolites, and/or irradiation as well as lenalidomide (9, 10). Hematological SPM represent serious, often life-threatening late complications arising in patients previously treated with high-dose chemotherapy and auto-HCT (11).

With significant improvements in MM therapies and prolonged survival, the incidence of secondary neoplasms has increased, drawing clinical attention to their management and outcomes (12–14). In this context, allogeneic HCT (allo-HCT) remains an option with curative intent to tackle secondary blood disorders after MM treatment, offering a graft-versus-malignancy effect that may overcome resistance to conventional therapies. At the same time, allo-HCT could possibly cure multiple myeloma as underlying disease, a condition otherwise deemed incurable until recently (15, 16). However, evidence guiding its implementation specifically in MM-associated t-HM is limited, stemming largely from retrospective series and registry analyses (12, 17). For instance, a European Society for Blood and Marrow Transplantation (EBMT) registry study evaluating allo-HCT in patients with treatment-related myeloid neoplasms (t-MN) arising after MM therapy reported that, although non-relapse mortality (NRM) and t-MN relapse remain challenges, allo-HCT yields long-term survivorship in a substantial subset of patients (17). Similarly, institutional cohorts suggest that allo-HCT may achieve sustained remission and meaningful survival outcomes, even in the context of adverse cytogenetics or high-risk disease biology (10, 12, 18). The reported cumulative risk of t-HM for MM patients receiving auto-HCT has varied widely across studies (19, 20). Historically, the development of t-HM has been associated with significant morbidity and poor survival due to high-risk cytogenetics and frequent *TP53* mutations, however treatment options for acute leukemias and MDS have evolved over time (6, 13, 18, 21).

This study aims to determine the prevalence of t-HM in a historical MM cohort and to investigate the clinical features and treatment outcomes of t-HM patients who were subjected to allo-HCT compared to their counterparts who did not proceed to allo-HCT.

## Methods

We retrospectively reviewed the medical records of all patients ≥ 18 years of age who received a 1^st^ auto-HCT for MM in our institution between 01 January 2010 and 30 April 2025 and later developed a t-HM. All patient charts were manually screened and patients who subsequently developed acute lymphoid or myeloid leukemia as defined by the 2016 World Health Organization classification (22), or t-MDS, were included within this study. This was an institutional review board-approved (UMG IRB 15/6/25), single-institution analysis and was conducted in accordance with the ethical principles of the Declaration of Helsinki. Patient demographics, molecular and cytogenetic analyses, treatments received, and clinical outcomes were obtained and reviewed from the electronic medical record in accordance with the University Medical Center Goettingen Institutional Review Board.

Median and ranges were calculated to describe patients’ characteristics. Baseline characteristics were assessed via independent t-test, Fisher’s exact test, and 2-sided Pearson chi-square analysis as appropriate. A Log-Rank test was performed to compare outcomes of allo-HCT recipients vs. non-recipients. Survival analysis was conducted using Kaplan-Meier methodology. Follow-up after allo-HCT was calculated using the reverse Kaplan–Meier method, with allo-HCT as the starting point and death or last follow-up as the end of observation. Overall survival (OS) and progression free survival (PFS) were censored at date of last follow-up.

Cumulative incidences of NRM and relapse-related mortality (RM) following allo-HCT were estimated in a competing-risk framework using the Aalen-Johansen estimator. Time was calculated from the date of allo-HCT to death or last follow-up. NRM and RM were treated as mutually competing events, while patients alive at last follow-up were censored. NRM was defined as death without RM, whereas RM was defined as death attributed to disease relapse. Cumulative incidence estimates were reported at day +100, day +200, 1 year, and 2 years after allo-HCT. Causes of death were additionally evaluated descriptively and categorized as relapse-related mortality, infection-related mortality, or other NRM based on the documented cause of death. Given the limited sample size, analyses were considered exploratory.

Given the limited number of observed deaths (n=11), multivariable Cox regression was not performed. Inclusion of multiple covariates in relation to the small number of events would have resulted in a low events-per-parameter ratio, increasing the risk of model overfitting and unstable effect estimates. Therefore, Cox regression analyses were restricted to exploratory univariable models.

A *P* value of <0.05 was deemed significant. All analyses were performed using R statistical software version 4.2.2 (available online at http://www.R-project.org).

For NGS analysis, genomic DNA was extracted from patient samples following cell separation by lysis. DNA libraries were prepared using the QIAseq Targeted DNA Kit (QIAGEN, Hilden, Germany), targeting the genes and exons specified in the respective panel. High-throughput sequencing was performed on Illumina MiniSeq or NextSeq platforms. Bioinformatic analysis included sequence alignment and variant identification, including single-nucleotide variants and small insertions and deletions (indels). The analytical limit of detection for mutation assessment was 5% variant allele frequency (VAF). For longitudinal analyses and hotspot mutations, a lower detection threshold of 1% VAF was applied.

## Results

### Myeloma patient baseline characteristics

At our institution, 519 patients underwent their first auto-HCT for MM during the specified time period. Of this cohort, 23 patients (4.4%) developed t-HM after exposure to myeloma-directed agents. Clinical and pathologic characteristics of these MM patients are summarized in **Table 1**. The median age at MM diagnosis was 57.4 years (42.5-74.3) and 7 patients (30%) were diagnosed with high-risk cytogenetics (17p deletion, t(4;14), t(14;16), t(14;20), 1q gain). All 23 patients (100%) received proteasome inhibitor-containing induction therapy prior to 1^st^ auto-HCT and the majority was exposed to alkylating agents (n= 18; 78%), aside from stem cell mobilization and high-dose conditioning before auto-HCT. Of note, 22/23 (96%) patients received cyclophosphamide chemotherapy alone or in combination with etoposide as stem cell mobilization regime before 1^st^ auto-HCT. Median time from MM diagnosis to 1^st^ auto-HCT was calculated at 6.6 months (4.0-18.8), with melphalan as alkylating agent being the ubiquitous conditioning regimen in all 23 cases. Median age at 1^st^ auto-HCT was 58 years. 14/23 patients (61%) received a 2^nd^ auto-HCT for high-risk genetics or in relapse, while one patient (4%) proceeded to a 3^rd^ auto-HCT. Likewise, these auto-HCT were preceded by melphalan-based conditioning. Of note, 19 patients (83%) received maintenance therapy with the immunomodulatory compound lenalidomide. The median number of therapy lines before t-HM diagnosis was two (1-5) and the median follow-up of surviving patients from MM diagnosis was calculated at 91.2 months (52.4-149.5). Baseline characteristics stratified by allo-HCT status are provided in **Supplementary Table 1**.

**Table 1:** Clinical and pathologic characteristics of MM patients that developed t-HM.

Characteristics	t-HM (n= 23)
Age at MM diagnosis (years), median (range)	57.4 (42.5-74.3)
Gender, n (%)	
Female	9 (39%)
Male	14 (61%)
Myeloma subtype, n (%)	
IgG	12 (52%)
IgA	2 (9%)
Kappa	6 (26%)
Lambda	2 (9%)
Asecretory	1 (4%)
R-ISS stage at diagnosis, n (%)	
I	3 (13%)
II	12 (52%)
III	4 (17%)
Missing	4 (17%)
High-Risk Cytogenetics, n (%) (17p deletion, t(4;14), t(14;16), t(14;20), 1q gain)	7 (30%)
MM induction therapy before 1^st^ auto-HCT, n (%)	
Proteasome inhibitor	23 (100%)
IMiDs	4 (17%)
Alkylating agents	18 (78%)
Topoisomerase inhibitors	1 (4%)
Anti-CD38 antibody	2 (9%)
Radiation therapy	7 (30%)
Auto-HCT for myeloma	23 (100%)
Chemotherapy for stem cell mobilization before 1^st^ auto-HCT	
Cyclophosphamide	18 (78%)
Cyclophosphamide/Etoposide	4 (17%)
Etoposide only	1 (4%)
Disease status at 1^st^ auto-HCT, n (%)	
sCR	7 (30%)
CR	4 (17%)
vgPR/PR	9 (39%) (3 (13%)
MR/SD/PD	
Time from MM diagnosis to 1^st^ auto-HCT (mo), median (range)	6.6 (4.0-18.8)
Total number of auto-HCT, n (%)	
1	8 (35%)
2	14 (61%)
3	1 (4%)
Maintenance lenalidomide after auto-HCT, n (%)	19 (83%)
Allogeneic HCT for myeloma, n (%)	1 (4%)
CAR T-cell therapy for myeloma, n (%)	1 (4%)
Therapy lines before t-HM diagnosis, median (range)	2 (1-5)
Follow-up of surviving patients from MM diagnosis (mo), median (range)	91.2 (52.4-149.5)

MM, multiple myeloma; t-HM, therapy-related hematological malignancy; R-ISS, Revised International Staging System; auto-HCT, autologous hematopoietic cell transplantation; IMiDs, immunomodulatory drugs; (s)CR, stringent complete response; (vg)PR, very good partial response; SD, stable disease; PD, progressive disease; MR, minimal response; mo, months; CAR T-cell, chimeric antigen receptor T-cell; mo, months.

### Disease characteristics of t-HM

Clinical features and disease characteristics of t-HM after prior myeloma-directed therapy are presented in **Table 2**. The median latency period between 1^st^ auto-HCT and diagnosis of t-HM was 58.7 months (18.6-125.0) with a considerable range to development of the respective SPM, and the median latency between last auto-HCT and t-HM diagnosis was 44.0 months (15.4-111.8). The most common secondary blood cancer was t-AML (n= 10; 43%) followed by t-MDS (n= 8; 35%) and B-ALL (n= 4; 17%). The swimmer plot in **Figure 1** depicts the individual clinical course of patients from MM diagnosis until last follow-up after t-HM diagnosis with timepoint of auto-HCT for myeloma and allo-HCT for t-HM. Eight patients (35%) displayed complex cytogenetics. The most frequently mutated genes by next-generation sequencing (NGS) at the time of t-HM diagnosis were *TP53* (n= 12; 52%), *DNMT3A* (n= 7; 30%) and *RAS* (n= 4; 17%), respectively (**Table 2**). None of the four B-ALL patients had a *BCR::ABL* fusion. Detailed initial cytogenetic and molecular features of t-HM before therapeutic pressure are shown in **Supplementary Table 2**. Induction therapies for t-HM are summarized in **Table 2**. 6/15 patients with induction therapy received hypomethylating agents (40%). 14/23 patients (61%) went on to undergo consolidating allo-HCT in curative intent with a median age of 61.6 years (54.2-73.8).

**Table 2:** Disease characteristics of t-HM after myeloma treatment.

Clinical features	t-HM (n= 23)
Age at t-HM diagnosis (years), median (range)	62.1 (53.4-78.7)
Latency period between MM diagnosis and t-HM (mo), median (range)	68.2 (24.0-131.6)
Latency period between 1^st^ auto-HCT and t-HM (mo), median (range)	58.7 (18.6-125.0)
Latency period between last auto-HCT and t-HM (mo), median (range)	44.0 (15.4-111.8)
Myeloma disease status at time of t-HM diagnosis	
sCR	7 (30%)
CR	8 (35%)
vgPR/PR	5 (22%)
MR/SD/PD	3 (13%)
t-HM subtypes, n (%)	
t-AML	10 (43%)
AML-MRC	4 (17%)
t-MDS	8 (35%)
B-ALL	4 (17%)
*TP53*-Mutant Clonal Hematopoiesis	1 (4%)
Complex karyotype, n (%)	8 (35%)
Molecular aberrations by NGS analysis	
Epigenetic regulators	
*TET2*	3 (13%)
*DNMT3A*	7 (30%)
*BCOR*	2 (9%)
Chromatin regulation	
*ASXL1*	2 (9%)
Cell signaling	
*RAS pathway*	4 (17%)
Transcription factors	
*RUNX1*	3 (13%)
Tumor suppressor genes	
*TP53*	12 (52%)
*WT1*	2 (9%)
Induction therapy for t-HM (before allo-HCT), n (%)	15 (65%)
Induction therapy for t-HM, n (%)	
Hypomethylating agents	6 (40%)
Venetoclax	4 (27%)
CPX-351	7 (47%)
Cytarabine	1 (4%)
Topoisomerase inhibitors	1 (4%)
GMALL (elderly) protocol for B-ALL	4 (27%)
Response to induction, n (%)	
CR	6 (40%)
PR	5 (33%)
SD/PD/Refractory	4 (27%)
**Allo-HCT for t-HM, n (%)**	
Yes	14 (61%)
No	9 (39%)
Reasons for not proceeding to allo-HCT (n= 9)	
Sufficient disease control of t-HM with conventional therapy	3
Ineligibility (Age, frailty or poor performance status)	1
Death before allo-HCT/refractory disease	5

t-HM, therapy-related hematological malignancy; MM, multiple myeloma; mo, months; auto-HCT, autologous hematopoietic cell transplantation; t-AML, therapy-related acute myeloid leukemia; AML-MRC, acute myeloid leukemia with myelodysplasia-related changes; t-MDS, therapy-related myelodysplastic syndrome; B-ALL, B-cell acute lymphoblastic leukemia; NGS, next-generation sequencing; allo-HCT, allogeneic hematopoietic cell transplantation; GMALL, German Multicenter Study Group for Adult ALL; (s)CR, (stringent) complete response; vgPR, (very good) partial response; MR, minimal response; SD, stable disease; PD, progressive disease; FU, follow-up; OS, overall survival.

**Figure 1:**
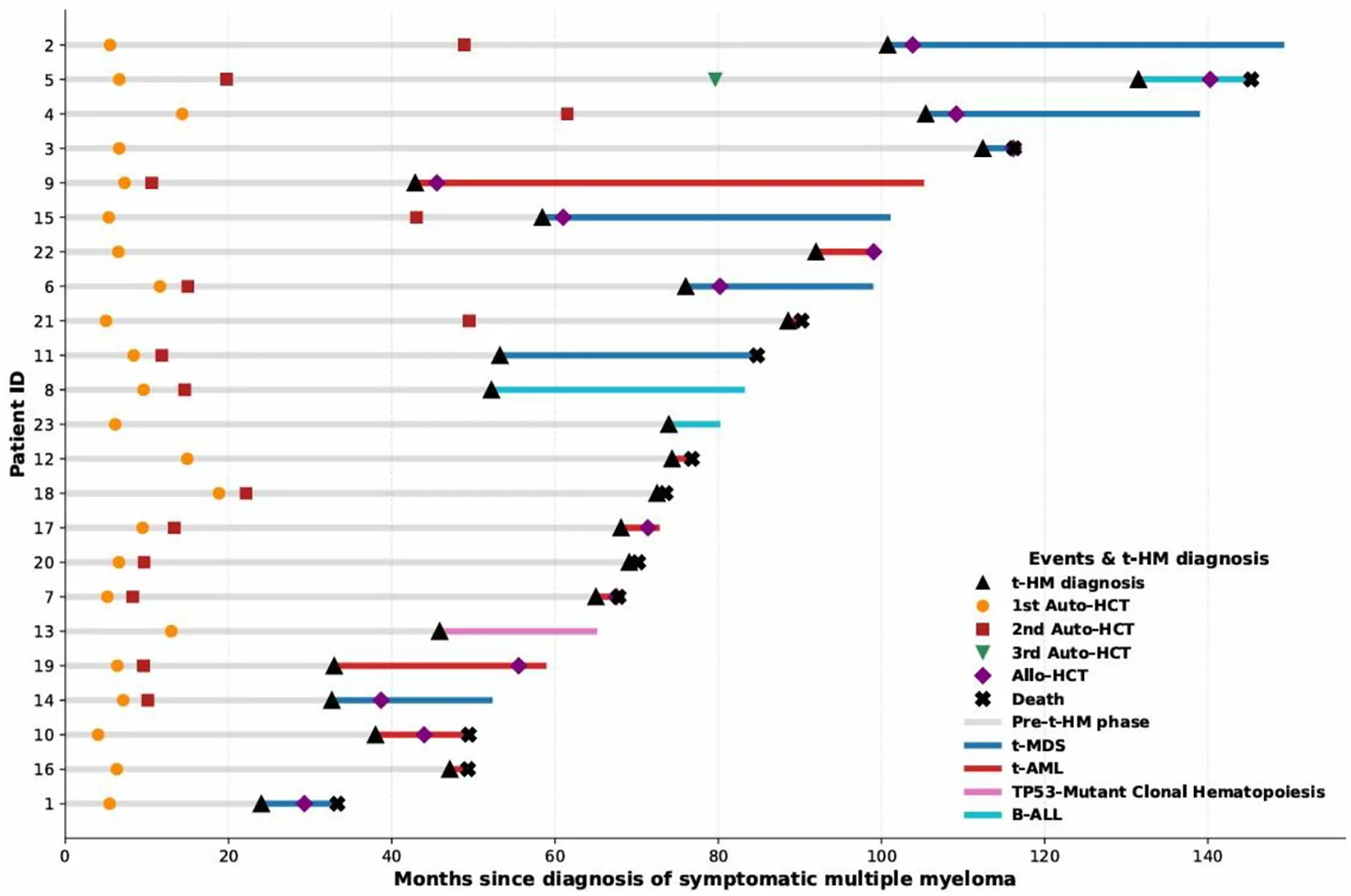
Swimmer plot illustrating patient-level timelines from first diagnosis of multiple myeloma. Horizontal bars represent follow-up from MM diagnosis. Symbols indicate respective autologous hematopoietic cell transplantation (auto-HCT), initial diagnosis of t-HM (blue triangle), timepoint of allo-HCT if applicable (purple rhombus). The different colors indicate respective subtype of t-HM. The black cross indicates death from any cause.

One patient (#13, **Figure 1**) developed CD34^+^ *ASXL1*- and *TP53*-mutant clones and was monitored closely and remained on lenalidomide maintenance therapy until last follow-up. The VAFs were measured at 15.2% (*ASXL1*) and 7.8% (*TP53*), respectively. Here, bone marrow morphology did not meet diagnostic criteria for MDS. Notably, one MM patient (patient #7, **Figure 1**) developed an AML with myelodysplasia-related changes (AML-MRC) with two *TP53* mutations (multi-hit) and a *DNMT3A* mutation plus complex karyotype following double auto-HCT, lenalidomide maintenance and CAR T-cell therapy with idecabtagene vicleucel. However, it is not known whether this patient had a preexisting clone that progressed after CAR-T in the bone marrow and showed clonal evolution before becoming clinically apparent as secondary AML (sAML). This patient underwent allo-HCT and died from neutropenic sepsis eight days afterwards.

### Efficacy and outcomes of allo-HCT

**Figure 2** shows the OS estimate for all 23 t-HM patients. Median OS for the entire cohort from time of t-HM diagnosis was calculated at 31.5 months, the median follow-up at 26 months. **Figure 3** highlights outcomes of allo-HCT recipients vs. non-recipients from t-HM diagnosis. The estimated median OS for allo-HCT recipients (not reached; 95% CI ≥ 9.4 months) was significantly better than for the nine non-transplanted patients with 2.5 months (95% CI 1.1-31.5 months), *P* = 0.0462. As depicted in **Figure 1**, five patients died shortly after allo-HCT, mainly from infections as major driver of non-relapse mortality. At data cut-off, 12/23 patients (52%) were still alive. Of the 14 allo-HCT patients, nine (64%) were alive at last follow-up while only 3/9 non-transplant recipients (33%) had survived their SPM for 31, 19 and 6 months, respectively (patients #8, #13 and #23, **Figure 1**).

**Figure 2:**
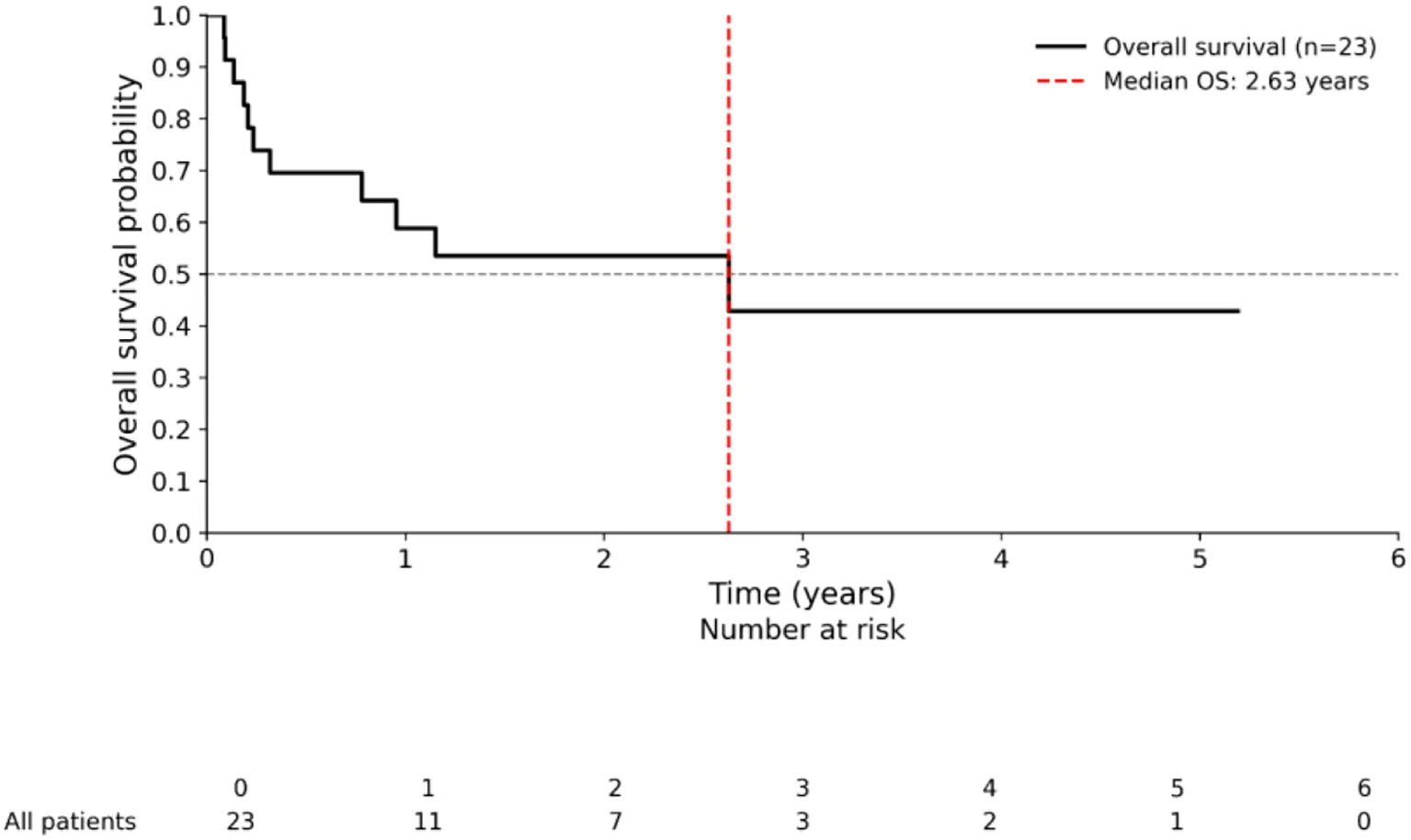
Kaplan-Meier for overall survival (OS) from time of t-HM diagnosis. Median OS for the entire cohort (23 patients) from time of t-HM diagnosis was calculated at 2.63 years.

**Figure 3:**
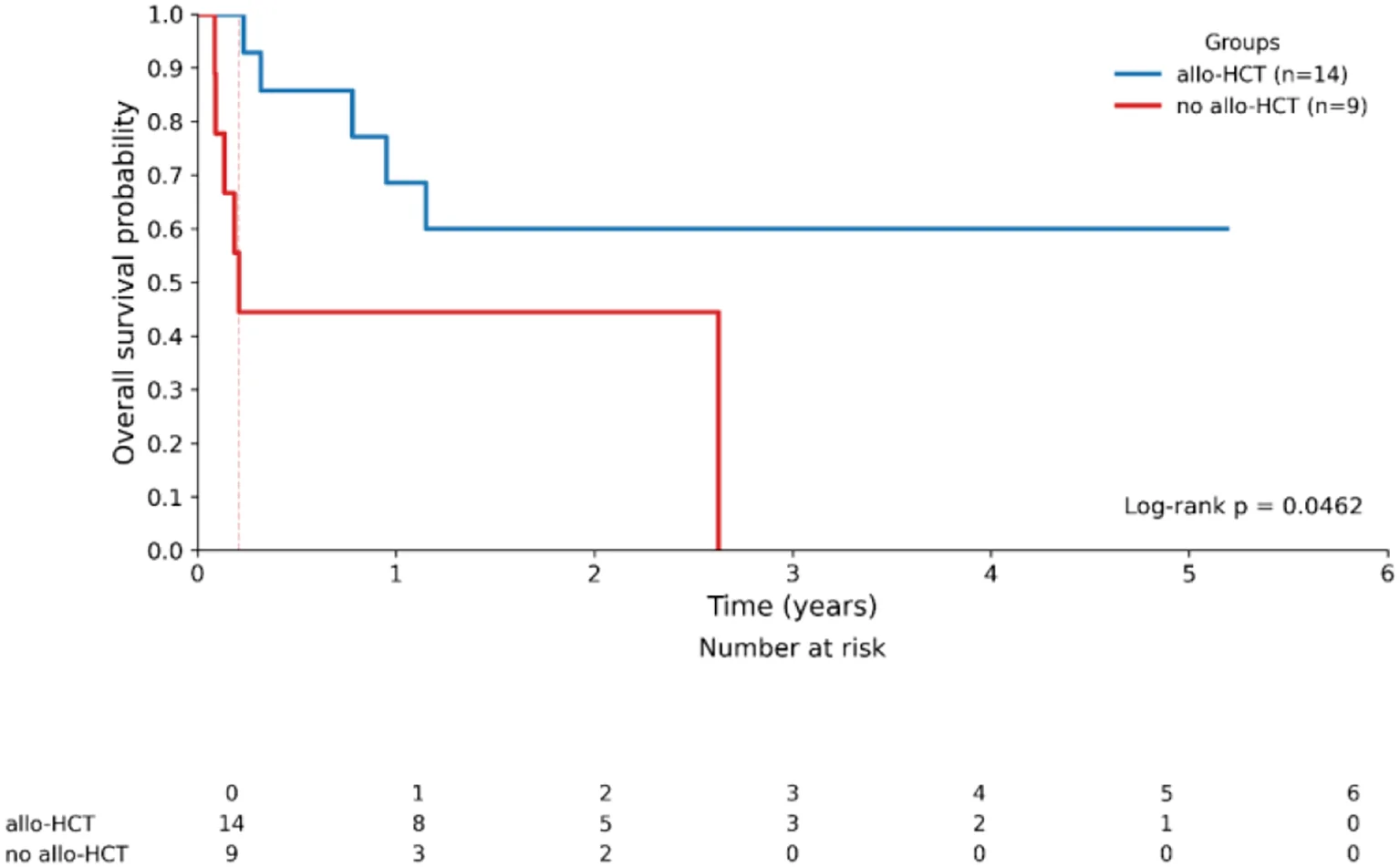
Overall survival probability for 23 t-HM patients by allogeneic hematopoietic cell transplantation status. A Log-Rank test was performed to compare outcomes of allo-HCT recipients vs. non-recipients. The estimated median OS for allo-HCT recipients (not reached; 95% CI ≥ 0.78 years) was significantly better than for the nine non-transplanted patients with 0.21 years (95% CI 0.09-2.63 years), P = 0.0462.

Allo-HCT characteristics are showcased in **Table 3**. Median time from t-HM diagnosis to allo-HCT was 4.0 months (2.5-22.6). Importantly, 10/14 patients (71%) received reduced-intensity conditioning. Patient #1 died from recurrent meningeal myeloma post-allo-HCT while patient #5 succumbed to relapsed/refractory (r/r) B-ALL post-allo-HCT. Patient #6 was administered five donor lymphocyte infusions (DLI) for serological recurrence of MM and reemergence of MDS phenotype in the bone marrow post-allo-HCT without loss of chimerism (LOC) and was alive and in remission at last follow-up. Median follow-up from allo-HCT was 29.8 months (0.1-59.8).

**Table 3:** Allo-HCT recipients and their transplant characteristics.

Clinical features	Allo-HCT recipients (n= 14)
Age at Allo-HCT (years), median (range)	61.6 (54.2-73.8)
Time from t-HM diagnosis to allo-HCT (mo), median (range)	4.0 (2.5-22.6)
Conditioning intensity, n (%)	
MAC	4 (29%)
RIC	10 (71%)
Donor type, n (%)	
Matched related donor (MRD)	5 (36%)
Matched unrelated donor (MUD)	6 (43%)
Mismatched unrelated donor (mMUD)	2 (14%)
Haploidentical donor	1 (7%)
Graft source, n (%)	
PBSC	14 (100%)
ATG use, n (%)	13 (93%)
GvHD prophylaxis	
Calcineurin inhibitor + MMF	13 (93%)
Post-transplant cyclophosphamide (PTCy)	1 (7%)
**GvHD occurrence, n (%)**	5 (36%)
aGVHD	4 (29%)
cGvHD	2 (14%)
DLI, n (%)	3 (21%)
Relapse incidence, n (%)	3 (21%)
Follow-up from Allo-HCT (mo), median (range)	5.3 (0.1-59.8)
Non-relapse mortality at 100 days, n (%)	2 (14%)
Survival status at last FU, n (%)	
Alive	12 (52%)
Dead	11 (48%)
Allo-HCT recipients: alive at last FU, n (%)	9 (64%)
No allo-HCT: alive at last FU, n (%)	3 (33%)
OS since t-HM diagnosis (mo), median	31.5
Follow-up of surviving patients from t-HM diagnosis (mo), median (range)	24.5 (4.7-62.4)
Follow-up from allo-HCT (mo), median	29.8 (0.1-59.8)

allo-HCT, allogeneic hematopoietic cell transplantation; t-HM, therapy-related hematological malignancy; mo, months; MAC, myeloablative conditioning; RIC, reduced-intensity conditioning; PBSC, peripheral blood stem cell; ATG, anti-thymocyte globulin; GvHD, graft-versus-host disease; DLI, donor lymphocyte infusion; MMF, Mycophenolate mofetil.

Among 14 patients undergoing allo-HCT, five deaths occurred during follow-up, including three NRM events and two relapse-related deaths. The cumulative incidence of NRM was 14.3% at day +100 and 23.8% at day +200, while no relapse-related deaths occurred within the first 100 days and the cumulative incidence of relapse-related mortality reached 19.0% at day +200. No additional deaths occurred thereafter, resulting in one- and two-year cumulative incidences of 23.8% for NRM and 19.0% for relapse-related mortality. Among the five deaths, two (40%) were relapse-related and one (20%) was attributed to infection; the remaining two (40%) were classified as other NRM.

In univariate Cox regression analyses starting from the diagnosis of therapy-related hematologic malignancy, none of the evaluated clinical variables was significantly associated with overall survival. A higher number of prior therapy lines showed a trend toward inferior survival (HR 1.60, 95% CI 0.94–2.72; *P* = 0.084).

## Discussion

In this retrospective single-center analysis, 23 of 519 patients (4.4%) who underwent first auto-HCT for MM subsequently developed a t-HM. The median latency from first auto-HCT to t-HM diagnosis was 58.7 months, highlighting that these secondary malignancies may emerge several years after intensive MM therapy and emphasize the importance of long-term survivorship care (12–14, 17–20). The majority of t-HM were t-AML or t-MDS, while B-ALL accounted for a smaller proportion. Overall, patients with t-HM had aggressive disease characteristics and poor outcomes, particularly those who did not proceed to allo-HCT. The observed proportion of patients developing t-HM in our cohort is within the range reported in previous studies, although direct comparisons are limited by differences in follow-up duration, patient selection, treatment exposure, and definitions of secondary malignancies (23–26).

The biological characteristics of t-HM in our cohort further support their high-risk nature. *TP53* mutations were detected in 52% of patients, and 35% had complex cytogenetics, findings consistent with the adverse molecular and cytogenetic profile previously described in therapy-related myeloid neoplasms (6, 13, 18, 21). The frequent detection of mutations associated with clonal hematopoiesis, particularly *DNMT3A*, together with the findings in individual patients with *TP53*- or *ASXL1*-mutated clones, is compatible with a multistep model of leukemogenesis in which pre-existing or treatment-selected hematopoietic clones may acquire additional abnormalities before progression to overt t-HM (**Figure 4**) (27). Importantly, our retrospective analysis cannot establish a causal relationship between any individual MM treatment and the development of t-HM. Nevertheless, the high prevalence of prior exposure to alkylating agents, including melphalan and cyclophosphamide, and to lenalidomide is consistent with previous studies linking cumulative treatment exposure to an increased risk of hematological SPM (7, 28–35). In particular, lenalidomide maintenance has been associated with an increased incidence of hematological SPM in randomized trials and subsequent analyses (28–32, 36–39). Experimental and clinical data further suggest that lenalidomide may exert selective pressure on pre-existing *TP53*-mutated hematopoietic clones, providing a potential biological mechanism for the development of *TP53*-mutated t-MN (7, 40–44). However, the established survival benefit of lenalidomide maintenance generally outweighs the absolute risk of SPM, and the potential contribution of lenalidomide must be considered in the context of the substantial prior exposure to high-dose melphalan, other alkylating agents, and auto-HCT in this population (25, 33, 34).

**Figure 4:**
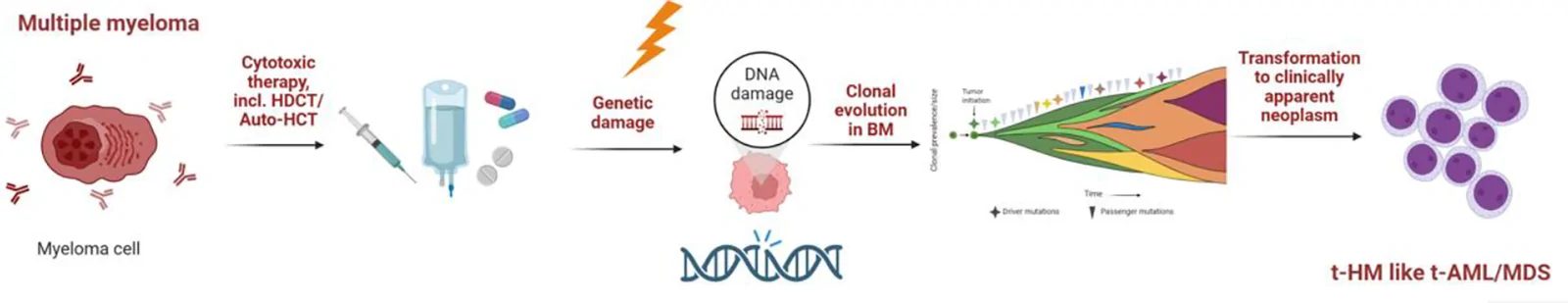
Proposed mechanism of genotoxic stress in the stem cell niche in myeloma patients making them susceptible to second primary malignancies (SPM). Therapy-related hematological malignancies (t-HM) may occur with considerable latency after accumulation of genotoxic stress during multiple lines of myeloma therapy. Alkylating agents and high-dose chemotherapy (HDCT) with melphalan before auto-HCT may especially contribute to clonal evolution in the stem cell nice of the bone marrow (BM). Image created with BioRender.com.

In our institutional cohort, fourteen of 23 patients (61%) underwent allo-HCT for t-HM. In an exploratory unadjusted analysis, patients who underwent allo-HCT had longer observed OS from t-HM diagnosis than those who did not undergo transplantation. This observation is consistent with previous institutional and registry-based reports suggesting that allo-HCT can provide durable disease control and long-term survival in selected patients with therapy-related myeloid neoplasms arising after MM treatment (12, 17, 18). However, the observed survival difference in our cohort should be interpreted with considerable caution. Patients proceeding to allo-HCT are inherently selected for younger age, better performance status, lower comorbidity burden, adequate organ function, disease control, and sufficient clinical fitness. In addition, allo-HCT was performed a median of 4.0 months after t-HM diagnosis and therefore represents a post-baseline treatment. Patients had to survive and remain eligible for transplantation in order to receive allo-HCT, introducing potential immortal-time bias into a conventional comparison of transplanted and non-transplanted patients. Accordingly, our findings should be interpreted as an association between allo-HCT and longer observed survival rather than as evidence of a definitive causal survival benefit. Nevertheless, the fact that a substantial proportion of selected patients remained alive following allo-HCT despite the adverse molecular characteristics of their t-HM supports consideration of allo-HCT as a potentially curative strategy in appropriate candidates. The EBMT experience similarly demonstrated that allo-HCT can achieve meaningful long-term survival in patients with t-MN after MM, although relapse and NRM remain important limitations (17).

The treatment landscape of MM has evolved substantially during the period covered by this study. CAR T-cell therapies and bispecific antibodies have become important treatment options in relapsed/refractory MM and are increasingly being introduced into earlier lines of therapy (45–48). These developments may alter the future epidemiology of SPM and t-HM in several ways. Reduced reliance on prolonged conventional cytotoxic therapy could potentially modify cumulative genotoxic exposure, while the increasing survival of patients with multiply relapsed MM may provide more opportunity for late complications to become clinically apparent. At the same time, secondary malignancies, including hematological malignancies and solid tumors, have been reported after commercial CAR T-cell therapy, although the absolute risk appears low and only a subset of reported hematological malignancies have demonstrated integration of the CAR transgene into the malignant clone (49–55). It therefore remains unclear whether the incidence, biology, and spectrum of SPM following contemporary MM treatment will differ from those observed in patients predominantly exposed to high-dose melphalan, other alkylating agents, and prolonged immunomodulatory therapy. Long-term follow-up of patients treated with CAR T-cell therapies and bispecific antibodies will be required to address this question.

Several limitations should be considered when interpreting our findings. First, the retrospective single-center design and small sample size of 23 patients limit statistical power and generalizability. Second, the comparison between allo-HCT recipients and non-recipients is inherently susceptible to selection bias and potential immortal-time bias, and therefore cannot establish a causal treatment effect. Third, the median follow-up after allo-HCT was only 29.8 months, limiting the maturity of post-transplant outcome estimates. Fourth, treatment strategies for both MM and t-HM evolved considerably over the 15-year observation period, potentially affecting the incidence, biology, and management of t-HM. Furthermore, our study was not designed to determine the relative contribution of individual anti-myeloma therapies to t-HM development, and solid tumor SPMs were not systematically assessed. Despite these limitations, the detailed clinical, cytogenetic, and molecular characterization of this uncommon patient population provides clinically relevant insights. The findings emphasize the importance of long-term surveillance of MM survivors, particularly those with substantial prior exposure to genotoxic therapies, and support consideration of longitudinal monitoring for clonal hematopoiesis in selected patients. For patients who develop t-HM, early multidisciplinary assessment of disease biology, response to initial therapy, comorbidities, and transplant eligibility may facilitate timely consideration of potentially curative treatment strategies, including allo-HCT.

## Conclusions

In MM, t-HM after high-dose chemotherapy/auto-HCT and lenalidomide maintenance are challenging and may occur with considerable latency. These t-HM are frequently associated with aggressive disease characteristics, e.g. high-risk cytogenetics and *TP53* mutations. Survival outcomes remain poor, however patients who underwent allo-HCT had better observed survival than patients who did not undergo allo-HCT. Surveillance and screening for clonal hematopoiesis might be useful for early detection of blood and marrow disorders potentially attributable to myeloma therapy.

## Data Availability

The datasets presented in this study can be found in online repositories. The names of the repository/repositories and accession number(s) can be found in the article/**Supplementary Material**.
