# Supplementary material for: Allogeneic hematopoietic cell transplantation for therapy-related hematological malignancies in patients with multiple myeloma
Source: Front Oncol. 2026 Sep 25;16:1887912. doi: 10.3389/fonc.2026.1887912 (PMC13649491; doi:10.3389/fonc.2026.1887912)
Supplement: Supplementary file 1 [file DataSheet1.docx]

**Supplemental table 1.**

**Baseline characteristics of MM patients stratified by allo-HCT status.**

| **Characteristics** | **Allo-HCT (n= 14)** | **No allo-HCT (n= 9)** |
| --- | --- | --- |
| Age at MM diagnosis (years), median (range) | 56.1 (42.5-67.7) | 62.7 (51.1-74.3) |
| **Gender, n (%)**  Female  Male | 2  12 | 7  2 |
| **Myeloma subtype, n (%)**  IgG  IgA  Kappa  Lambda  Asecretory | 6  2  5  1  0 | 6  0  1  1  1 |
| **R-ISS stage at diagnosis, n (%)**  I  II  III  Missing | 3  6  4  1 | 0  6  0 3 |
| High-Risk Cytogenetics, n (%)  (17p deletion, t(4;14), t(14;16), t(14;20), 1q gain) | 3 | 4 |
| **MM induction therapy before 1^st^ auto-HCT, n (%)**  Proteasome inhibitor  IMiDs  Alkylating agents  Topoisomerase inhibitors  Anti-CD38 antibody  Radiation therapy | 14  2  11  1  1  4 | 9  2  7 0 1 3 |
| Auto-HCT for myeloma | 14 | 9 |
| **Chemotherapy for stem cell mobilization before 1^st^ auto-HCT**  Cyclophosphamide  Cyclophosphamide/Etoposide  Etoposide only | 12  2  0 | 6  2  1 |
| **Disease status at 1^st^ auto-HCT, n (%)**  PR or better (vgPR, CR)  MR/SD/PD | 11  3 | 9  0 |
| Time from MM diagnosis to  1^st^ auto-HCT (mo), median (range) | 6.6 (4.0-14.3) | 8.4 (5.0-18.8) |
| Age at 1^st^ auto-HCT (years), median (range) | 56.5 (43.0-68.2) | 64.0 (51.6-75.0) |
| **Conditioning therapy for 1^st^ auto-HCT**  Melphalan | 14 | 9 |
| **Total number of auto-HCT, n (%)**  1  2  3 | 4  9  1 | 4  5  0 |
| Maintenance lenalidomide after auto-HCT, n (%) | 13 | 6 |
| Allogeneic HCT for myeloma, n (%) | 1 | 0 |
| CAR T-cell therapy for myeloma, n (%) | 1 | 0 |
| Therapy lines before t-HM diagnosis, median (range) | 3 (1-5) | 2 (1-4) |

MM, multiple myeloma; t-HM, therapy-related hematological malignancy; R-ISS, Revised International Staging System; auto-HCT, autologous hematopoietic cell transplantation; IMiDs, immunomodulatory drugs; CR, complete response; (vg)PR, (very good) partial response; SD, stable disease; PD, progressive disease; MR, minimal response; mo, months; CAR T-cell, chimeric antigen receptor T-cell; mo, months.
