# Supplementary material for: Allogeneic hematopoietic cell transplantation for therapy-related hematological malignancies in patients with multiple myeloma
Source: Front Oncol. 2026 Sep 25;16:1887912. doi: 10.3389/fonc.2026.1887912 (PMC13649491; doi:10.3389/fonc.2026.1887912)
Supplement: Supplementary file 2 [file DataSheet2.docx]

**Supplemental table 2.**

**Initial cytogenetic and molecular features of 23 selected t-HM cases.**

| **Patient #** | **Available initial cytogenetic and molecular genetic features of t-HM**  Corresponding variant allele frequencies (VAF) for somatic mutations are shown in [%] |
| --- | --- |
| **#1** | ISCN:  46, XY,del(20)(q11.2q13.2)[7]/ 46,XY[8] |
| **#2** | ISCN:  45,XY,der(2)t(2;13)(q3?7;q3?2),del(5)(q15q33),der(7)t(7;7)(p15;?p13),7,13,+mar,+mar[14]/45,idem,der(4)t(4;12)(q32;p13)del(4)(q21q32),der(12)t(4;12)(q32;p13)[3]/45,X,-Y,der(2)t(2;13)(q3?7;q3?2),-5,der(6)t(5;6)(p23;?p14)[4]/46,XY[1]  FISH:   - 12p deletion in 96% of nuclei - 13q14 deletion in 99% of nuclei - 5q deletion in 100% of nuclei - Y loss in 99% of nuclei - Monosomy 7 in 97% of nuclei   *TP53* (p.R282P): 46.2%  *TP53* (p.H193Y): 46.7%  *TET2* (VAF n/a) |
| **#3** | *NRAS* (p.G13D): 33.8%  *TP53* (p.C176G): 17.9%  *TP53* (p.N131T): 8.9% |
| **#4** | *BCOR* (D1581H): 52.0%  *TP53* (G334A): 26.3%  *TP53* (n/a): 22.2%  *U2AF1* (Q157P): 10.5%  Complex aberrant karyotype (13q-, 5q-, 7q-) |
| **#5** | ISCN:  47, XY,del(20)(p1?2)del(q12),+21[7]/46,XY[4]  FISH:   - Trisomy 21/*RUNX1* rearrangement in 84% of nuclei - 20q deletion in 91% of nuclei - No *BCR-ABL* rearrangement |
| **#6** | 4q12 deletion, 4q24 deletion, 5q deletion, 6q23 gain, 9p gain, 9q deletion, *RUNX1* amplification, *TET2* deletion, trisomy 21, partial trisomy 7, 7q deletion  *NRAS* (p.Q61R): n/a  *TP53 (*p.K132M): 46%  *TP53* (p.I195T): 46.1% |
| **#7** | ISCN:  45~47, XX,-7,+8,der(16)t(16;19)(p1?3;p13),der(19)r(19)(p13q13)hsr  (19)(p13),der(19)t(7;19)(q31;p13),dup(21)(12q13),der(21;22)(q10;q10)dup(21)  (q11q22),+dic(19;21)(p13;q22),+mar[cp22]  Complex aberrant karyotype (7q-, trisomy 21, 19p+)  FISH:  BM:   - 19p gain in 13% - 7q deletion in 32% - Trisomy 21/ *RUNX1* rearrangement in 13-21% of nuclei   PB:   - 7q deletion in 33% - Trisomy 21/ *RUNX1* rearrangement in 26% - Monosomy 7 in 11% of nuclei   *DNMT3A* (p.R499H): 3.5%  *TP53* (c.376-1G>A): 13.5%  *TP53* (p.R248W): 11.7% |
| **#8** | ISCN:  46, XX,del(9)(p13p2?2)[5]/ 46,XX[16]  - No *BCR-ABL* rearrangement  - 9p deletion |
| **#9** | Mutations in *WT1*, *NOTCH1*, *ETV6* |
| **#10** | ISCN:  nuc ish 7cen(D7Z1x2),7q31(D7S522x1)[16/106], 7cen(D7Z1x2),7q31(D7S522x2)[50/106], 12p13.2(TELx2),21q22(RUNX1x3)  [50/124], 1p32(CDKN2Cx2),1q21(CKS1Bx2)[104], 4q12(SCFD2x2),4q24(TET2x2)[100], 5p15.2(D5721,D5S23x2),5q31(EGR1x2)  [100], 8cen(D8Z2x2)[100], 11q23(5'KMT2Ax2,3'KMT2Ax2)(5'KMT2A con 3'KMT2Ax2)[100], 13q14(D13S319x2),13q34(LAMPx2)  [100], 17p13(TP53x2),17q11(NF1x2)[395], 20q12(D20S108x2)[100], Xcen(DXZ1x2)[100]  FISH:   - 7q deletion in 15% of nuclei - Trisomy 21/*RUNX1* rearrangement in 40% of nuclei - Monosomy 7 in 47% of nuclei   *CUX1* (p.A333T): 26.8%  *DNMT3A* (p.R693H): 36.1%  *RUNX1* (p.R204Q): 39.5%  *TET2* (p.P1460fs): 37.6%  *TET2* (p.Q1534X): 37.1% |
| **#11** | ISCN:  Nuc ish 1p32(CDKN2Cx2),1q21(CKS1Bx4)[26/126], 5p15.2(D5721,D5S23x4),5q31(EGR1x2)[21/105], 4q12(SCFD2x2),4q24  (TET2x2)[131], 7cen(D7Z1x2),7q31(D7S522x2)[114], 8cen(D8Z2x2)[105], 11q23(5'KMT2Ax2,3'KMT2Ax2)(5'KMT2A con 3'KMT2Ax2)  [102], 12p13.2(TELx2),21q22(RUNX1x2)[118], 13q14(D13S319x2),13q34(LAMPx2)[122], 17p13(TP53x2),17q11(NF1x2)[414], 20q12  (D20S108x2)[113], Xcen(DXZ1x2)[230]  FISH:   - Trisomy 1q - Partial tetrasomy 1q in 44% of nuclei - Tetrasomy 5p in 49% of nuclei   *DNMT3A* (p.S337L): 5.7%  *PPM1D* (p.M521fs): 14.9% |
| **#12** | ISCN: 43,XX,der(7;15)(q10;q10),del(7)(q?11.2q36),t(8;11)(q2?3;q2?2),der(9)t(9;19)(q1?3;q12),11,der(12)t(12;13)(p13;q12),der(12)t(12;18)(q2?4;q1?2),13,der(18)t(1;18)(?p36;q2?3),del(18)(q1?2q23),der(19)t(11;19)(q1?3;q12)[6]  FISH:   - Monosomy 5 (10%, 20%) - 13q34 deletion (10%, 17%) - 3q26 gain/3q26 rearrangement (6%, 20%) - 7q deletion (74%, 62%)   *TP53* (p.R196X): 33.9%  *TP53* (p.T123fs): 18.4%  *CEBPA* (p.R47fs): 6.1%  *DNMT3A* (p.D271fs): 32.7% |
| **#13** | ISCN:  46, XX[4]  nuc ish 1p32(CDKN2Cx2),1q21(CKS1Bx2)[106], 4q12(SCFD2x2),4q24(TET2x2)[97], 5p15.2(D5721,D5S23x2),5q31(EGR1x2)  [115], 7cen(D7Z1x2),7q31(D7S522x2)[107], 8cen(D8Z2x2)[109], 11q23(5'KMT2Ax2,3'KMT2Ax2)(5'KMT2A con 3'KMT2Ax2)[100],  12p13.2(TELx2),21q22(RUNX1x2)[103], 13q14(D13S319x2),13q34(LAMPx2)[95], 17p13(TP53x2),17q11(NF1x2)[393], 20q12  (D20S108x2)[109], Xcen(DXZ1x2)[100]  *ASXL1* (T639fs): 15.2%  *TP53* (R273H): 7.8% |
| **#14** | FISH:   - 1p32 deletion - 1q21 gain - 13q14 deletion - 17p deletion   *ASXL1* (p.R625fs): 24.7%  *ASXL1* (p.L823): 1.4%  *DNMT3A* (p.C562Y): 31.6%  *DNMT3A* (p.V636M): 1.8%  *TP53* (p.Y163C): 1.2% |
| **#15** | ISCN:  46, XY[4];  nuc ish 1p32(CDKN2Cx2),1q21(CKS1Bx2)[100], 4q12(SCFD2x2),4q24(TET2x2)[100], 5p15.2(D5721,D5S23x2),5q31  (EGR1x2)[100], 7cen(D7Z1x2),7q31(D7S522x2)[100], 8cen(D8Z2x2)[100], 11q23(5'KMT2Ax2,3'KMT2Ax2)(5'KMT2A con  3'KMT2Ax2)[100], 12p13.2(TELx2),21q22(RUNX1x2)[100], 13q14(D13S319x2),13q34(LAMPx2)[100], 17p13(TP53x2),17q11(NF1x2)  [385], 20q12(D20S108x2)[95], Xcen(DXZ1x2)[100]  *ASXL1* (p.H630fs): 19.3%  *TET2* (p.R1161fs): 42.9% |
| **#16** | ISCN:  46, XX,der(2)t(2;4)(3?5;2?2),t(3;21)(q26;q1?1.2)[10]/46,XX[29];  nuc ish (GATA2,RPN1x2,MECOMx3)[111/123], (SCFD2x2,TET2x3)[23/135], (D5721/D5S23,EGR1)x2[119], 7cen(7p11.1  q11.1x2),7q22(KMT2Ex2),7q31.2(D7S522/METx2)[130], 8cen(D8Z2x2),20q12(D20S108x2),20q13.3(RH74808/RH67654x2)[115],  8q21(RUNX1T1x2),21q22(RUNX1x2)[114], 11q23(5'KMT2Ax2,3'KMT2Ax2)(5'KMT2A con 3'KMT2Ax2)[138], 12p13.2(TELx2),21q22  (RUNX1x2)[133], (D13S319,LAMP1)x2[156], 15q24(PMLx2),17q21(RARAx2)[204], 16q22(5´CBFBx2,3´CBFBx2)(5´CBFB con 3  ´CBFBx2)[200], (TP53,NF1)x2[400], (DXZ1x2,DYZ1x0)[123]  *BCOR* (p.G665fs): 47.1%  *DNMT3A* (p.S105R): 49.6%  *IKZF1* (p.R213): 39.2%  *NRAS* (p.Q61R): 46.2%  *WT1* (p.H465fs): 40.9% |
| **#17** | ISCN:  46, XY,i(5](p10)[4]/46,XY,t(2;11)(q3?7;q1?3)[2]/46,XY[29]  *NRAS* (p.G12D): 2.6%  *TP53* (p.Y234C): 17.3%  *TP53* (p.V272M): 24.8% |
| **#18** | ISCN:  nuc ish 9q34(ASS1x2,ABL1x2),22q11.2(BCRx2)[227]   - No *BCR-ABL* rearrangement |
| **#19** | ISCN:  Nuc ish 1p32(CDKN2Cx2),1q21(CKS1Bx2)[100], 4q12(SCFD2x2),4q24(TET2x2)[100], (D5721/D5S23,EGR1)x2[100], 7cen  (7p11.1-q11.1x2),7q22(KMT2Ex2),7q31.2(D7S522/METx2)[100], 8cen(D8Z2x2),20q12(D20S108x2),20q13.3(RH74808/RH67654x2)  [100], 11q23(5'KMT2Ax2,3'KMT2Ax2)(5'KMT2A con 3'KMT2Ax2)[100], 12p13.2(TELx2),21q22(RUNX1x2)[100],  (D13S319,LAMP1)x2[100], (TP53,NF1)x2[418], (DXZ1,DYZ1)x1[100]  *RUNX1* (p.R162K): 11.7%  *TP53* (p.R273L): 31.5% |
| **#20** | ISCN:  45, XY,der(2)t(2;4)(q2?4;?q?),-5,der(6)t(6;20)(p?21;?q?13)t(6;5)  (q11;q?)t(5;8)(q?;?q2?1),del(7)(q11q36),der(20)t(6;20)(q11;q11)t(20;5)(p13;p11),dup(21)(q21q2?2)[21]  FISH:   - 5q deletion - 7q deletion - *RUNX1* loss - 20q deletion   *DNMT3A* (p.R882H): 39.2%  *TP53* (p.D393fs): 34.4%  *TP53* (p.A129fs): 42%  *TP53* (p.A129V): 40.6% |
| **#21** | ISCN:  44, XY, der (5) t(5;16)(q11;?q23), der (7;17)(p10;q10),der(11)(::11p11  >11q11::11q23->11q24::), del(13)(q14q31),der(14)(13qter->13q14::13q31  >13q21::14p11->14qter),-16,der(19)(10pter->19q11::11q14->11q25::19q11  >19q13::11q23->11qter) (cp6)  FISH:   - 5q31 deletion in 92% of nuclei - 7q31 deletion in 90% of nuclei - 17p deletion *(TP53*) in 88% of nuclei - Partial tetrasomy 11q in 12% of nuclei - Partial trisomy 11q in 33% of nuclei - 13q14 deletion/monosomy 13 in 21% of nuclei |
| **#22** | *RUNX1* mutation (c.239G>A) |
| **#23** | - 14q32 gain - CRLF2 deletion - No *BCR-ABL1* rearrangement |

VAF, variant allele frequency; ISCN, international system for human cytogenomic nomenclature; FISH, fluorescence in situ hybridization; BM, bone marrow; PB, peripheral blood.
